# A Family Exhibiting Autosomal Dominant Inheritance of Multiple Acyl-Coenzyme A (CoA) Dehydrogenase Deficiency (MADD) Disease

**DOI:** 10.3390/ijms27167294

**Published:** 2026-08-15

**Authors:** Francesco Baldo, Elena Genova, Valeria Capaci, Irene Marrone, Nour Balasan, Anna Monica Bianco, Luisa Zupin, Irene Bruno, Maria Teresa Bonati, Fulvio Celsi

**Affiliations:** 1 Division of Metabolic Diseases and Hepatology, Bambino Gesù Childrens Hospital IRCCS, 00165 Rome, Italy; francesco.baldo@opbg.net; 2 Institute for Maternal and Child Health—IRCCS “Burlo Garofolo”, 34137 Trieste, Italy; elena.genova@burlo.trieste.it (E.G.); valeria.capaci@burlo.trieste.it (V.C.); irene.marrone@studenti.units.it (I.M.); nour.balasan@burlo.trieste.it (N.B.); annamonicarosaria.bianco@burlo.trieste.it (A.M.B.); luisa.zupin@burlo.trieste.it (L.Z.); irene.bruno@burlo.trieste.it (I.B.); mariateresa.bonati@burlo.trieste.it (M.T.B.)

**Keywords:** electron transfer flavoprotein dehydrogenase (ETFDH), muscle weakness, riboflavin, fatty acid metabolism, autosomal dominant, primary fibroblasts

## Abstract

Multiple acyl-CoA dehydrogenase deficiency (MADD) is considered an autosomal recessive disorder; yet, recent findings suggest up to 10% of cases may result from heterozygous electron transfer flavoprotein dehydrogenase (*ETFDH*) variants exhibiting dominant or dominant-like effects. Here, a novel heterozygous *ETFDH* variant (c.1798A>C, p.Asn600His) was identified within a three-generation family. The grandfather presented with muscular weakness at age 35, and the father developed similar symptoms at 19 following a tonsillectomy. Both were diagnosed with MADD based on muscle biopsies revealing neutral lipid accumulation and acylcarnitine profiles and responded fully to riboflavin therapy (150 mg/day). The two siblings, aged 8 and 10, carry the same mutation and show increased acyl-carnitine levels but remain asymptomatic due to early riboflavin treatment. Skin fibroblasts from affected individuals were immortalized and subjected to normal and reduced riboflavin levels. Gene expression analysis demonstrated unchanged *ETFDH* RNA but reduced protein levels in mutant cells, particularly under low riboflavin. Structural modelling suggested the Asn600His substitution destabilizes the protein, diminishing its mitochondrial function. Proximity ligation assays indicated a decreased interaction with mitochondrial complex III, while oxygen consumption via fatty acid oxidation was impaired, especially at reduced riboflavin. The novel *ETFDH* variant found in this family gives a possible dominant pattern of inheritance for MADD, where a single mutant allele impairs the mitochondrial metabolism, particularly under riboflavin-deficient conditions, and highlights the importance of early riboflavin supplementation in preventing clinical symptoms.

## 1. Introduction

Electron Transfer Flavoprotein Dehydrogenase (ETF dehydrogenase—ETFDH—or electron transfer flavoprotein-ubiquinone oxidoreductase—ETFD:QO) is a key mitochondrial enzyme, and its dysfunction leads to multiple acyl-CoA dehydrogenation deficiency (MADD), an autosomal recessive congenital metabolic disorder [1]. Three clinical presentations of MADD are currently known: a severe form, that typically affects newborns and is usually fatal in the first weeks of life, characterized by metabolic acidosis, hypoglycaemia, hyperammonaemia with liver dysfunction, hypotonia, and congenital malformations (renal cysts) (type I); an infantile, less severe form, characterized by myopathy (muscle weakness, exercise intolerance, and muscle pain) and cardiac involvement, episodic hypoglycaemia and acidosis, and, possibly, neurological involvement (seizures, and encephalopathy) (type II); and a milder, late-onset form, characterized by myopathy and metabolic decompensation (type III) [2]. Although MADD is classically autosomal recessive, a meta-analysis indicates that some late-onset patients carry single heterozygous ETFDH variants. These variants might affect different residues on the protein’s sequence with a higher pathogenicity score, compared to biallelic variants [3].

**Figure 1:**
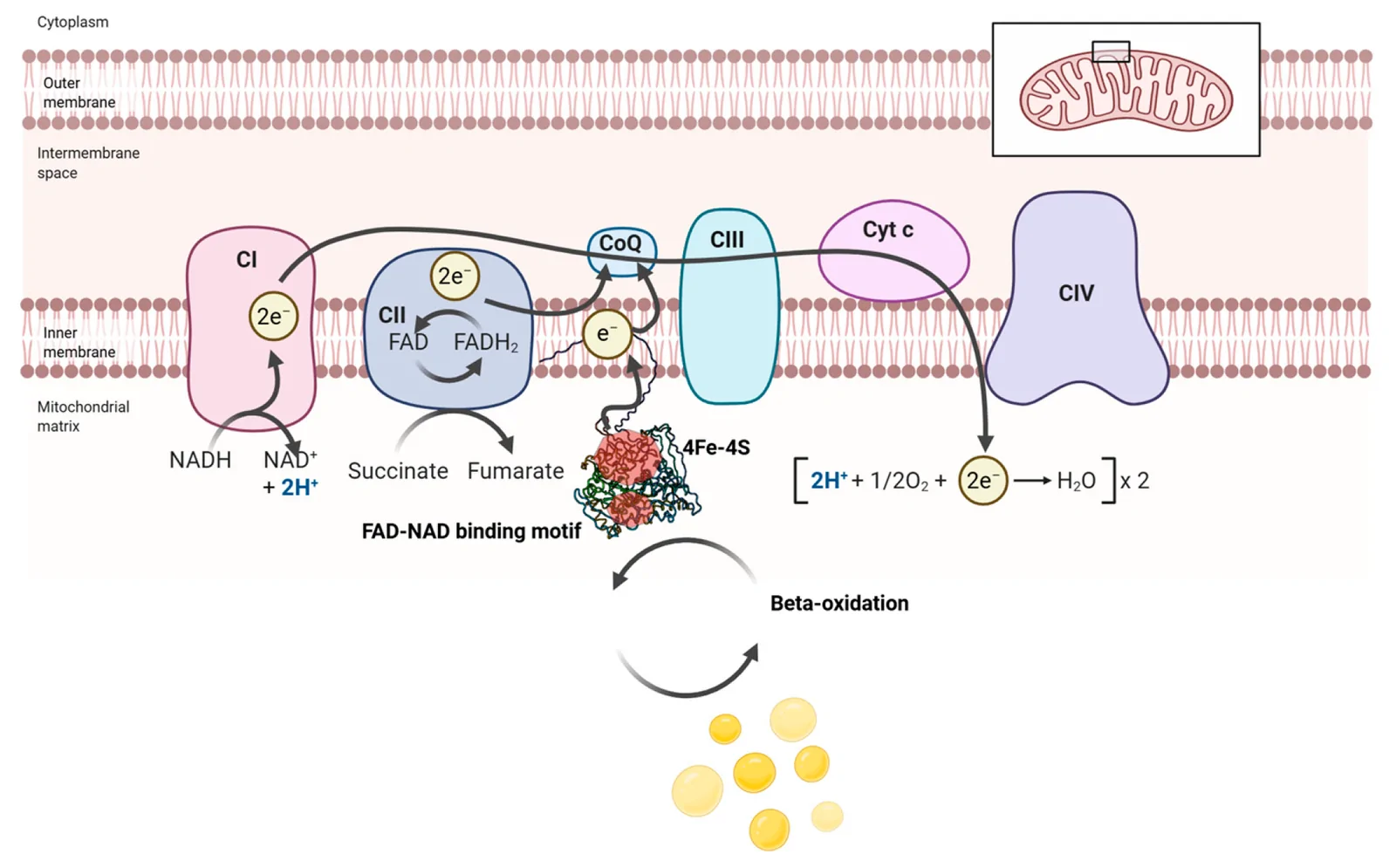
Diagram of ETFDH role in transferring electrons from the beta-oxidation cycle to Electron Transfer Chain in mitochondria. MADD mutations are principally localized in FAD-NAD binding domain and 4Fe-S cluster.

Specifically, ETFDH connects the beta-oxidation of fatty acids to the mitochondrial respiratory chain. The enzyme is a single protein embedded in the inner mitochondrial membrane, where it transfers electrons from oxidized fatty acids to ubiquinone (UQ) [4]. Structurally, ETFDH contains one molecule of flavin adenine dinucleotide (FAD), one 4Fe-4S cluster, and one UQ-binding site (Q site) [5]. The fatty acid oxidation spiral strips off two electrons during the formation of acetyl-CoA. Electrons are then transferred from ETFA-B to ETFDH, which, in turn, transfers them to UQ, reducing it to ubiquinol (UQH2). The reduction of UQ to UQH_2_ occurs in two steps: during the first round of the Q-cycle, one electron is transferred to reduce a first molecule of cytochrome c, while the second electron generates a semiquinone radical (UQH•); thereafter, a second cytochrome c is reduced and the semiquinone intermediate is stabilized and fully reduced to UQH_2_ at the level of the cytochrome b (Cytb) subunit of complex III (CIII) [5,6]. Specifically, ETFDH, CIII, and UQH2 are physically associated in a membrane complex in order to minimize electron leaks and the production of reactive oxygen species production [5]. By reducing two molecules of cytochrome c at the level of complex III derived from one acetyl-CoA generated by FAO, ETFDH plays a crucial role linking between fatty acids oxidation to ATP production (Figure 1) [7].

Currently, MADD treatment includes a low-fat, low-protein, high-carbohydrate diet and supplementation with carnitine (if deficient), coenzyme Q10, and riboflavin (vitamin B2), an essential cofactor for ETFDH activity. Indeed, ETFDH is a flavoprotein requiring FAD for its activity. Since FAD is derived from riboflavin, a deficiency in riboflavin can impair ETFDH function, either decreasing its stability [8] or negatively affecting its interaction with complex III and mitochondrial electron transfer efficiency in MADD patients. For this reason, riboflavin supplementation may be beneficial in a subset of patients. This study describes the effects, at the cellular level, of a familial ETFDH variant (NM_004453.4: c.1798A>C, p.Asn600His) not previously associated with MADD.

## 2. Results

### 2.1. Acylcarnitine Profiles

In this study, we report the biochemical profile of a family (grandfather, father, and two siblings; Supplementary Figure S1) in which the two older subjects (grandfather and father) display symptoms associated with MADD. In both the father and the siblings, riboflavin induced a dramatic change in the acylcarnitine pattern, with a complete normalization in the youngest sibling (Table 1).

### 2.2. Genetic Analysis

The grandfather, the father, and the two siblings underwent Whole Exome Analysis; the only variation linked to the pathology was identified on the gene ETFDH, while, on the other genes linked to beta-oxidation, no other putative variations were found. The ETFDH (NM_004453.4): c.1798A>C, p. (Asn600His) variant was classified as likely pathogenic according to the ACGS/ACMG-AMP criteria PM1_moderate, PP2_supporting, PM2_moderate, and PP3_supporting (https://franklin.genoox.com/clinical-db/home, accessed on 10 February 2026). This variant is present in population databases (rs770905850, gnomAD 0.003%) and has not been reported in the literature in individuals affected with ETFDH-related conditions.

Whole genome analysis in the father revealed no other putative nucleotide variations that could be causative of the disease. Interestingly, the inheritance pattern in the family appears to be autosomal-dominant, while MADD is a typical autosomal-recessive disorder.

### 2.3. ETFDH Variant Detection and Allelic Expression Analysis

To perform functional studies in vitro, we obtained primary skin fibroblasts from a punch biopsy and immortalized them by hTERT-Cdk4 viral transduction. The Sanger sequencing of cDNA confirmed the presence of the c.1798A>C (p.Asn600His) ETFDH variant in RNA from fibroblasts of all the three affected family members, while the variant was absent in the healthy control (Supplementary Figure S2), thus representing a good cellular model for downstream analysis.

To further assess if the inheritance pattern could be explained by allelic variance, digital PCR analysis further quantified the allelic expression, showing that the mutant and wild-type transcripts in fibroblasts from the patients were each expressed at approximately 50%, indicating no allele-specific transcript imbalance (Supplementary Figure S3).

### 2.4. Structural Analysis of ETFDH Protein

**Figure 2:**
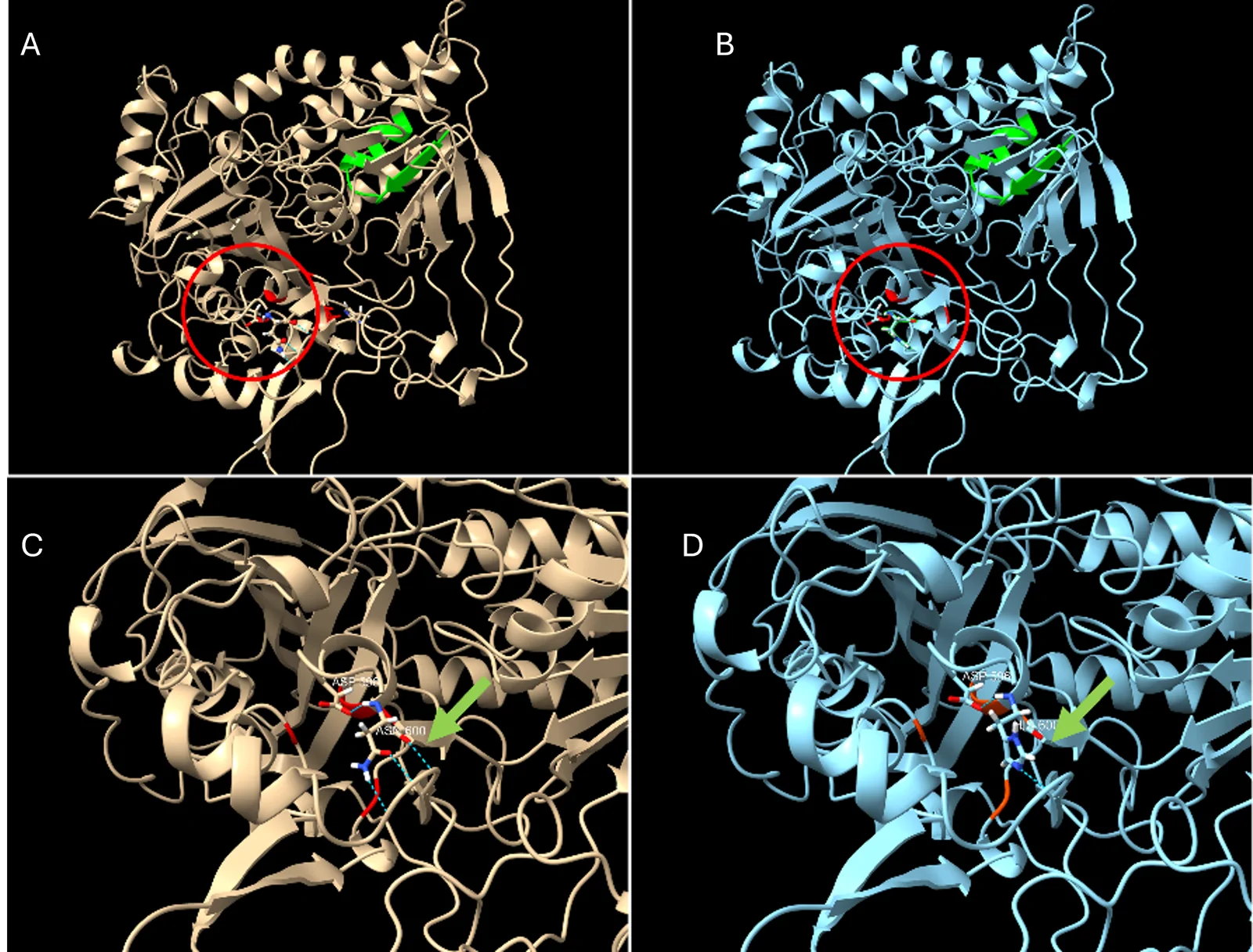
The three-dimensional structure of ETFDH wild-type (**A**–**C**) and p.Asn600His (**B**–**D**). (**A**,**B**) display the total structure of ETFDH, in green UQ-binding domain (Q-site), while in red are the key aminoacid for 4Fe-S domain. (**C**,**D**) display a focused image on 600 residue, with Asn being in (**C**) and His in (**D**). It is possible to observe the reduced number of hydrogen bonds (green arrow).

To evaluate the impact of the variation on the ETFDH structure, we exploited the AlphaFold 3 webserver to obtain a simulated three-dimensional structure of the protein (Figure 2) [9]. We observed that Asn600 is buried in the protein structure, near the 4Fe-4S motif and in contact with a structural loop (Figure 2A) using three hydrogen bonds (Figure 2C). The variant p.Asn600His, although not causing large structural changes, lowers the number of hydrogen bonds toward the loop (Figure 2B,D). The prediction from AlphaMissense [10] indicates this change as “pathogenic” with a score of 0.932 (1 being the maximum score for pathogenicity). The aminoacid is also highly conserved, present in mouse, rat, and drosophila (Supplementary Figure S4). This preliminary analysis indicates that the p.Asn600His change can influence protein stability and/or activity.

### 2.5. Riboflavin Supplementation and ETFDH Protein Expression

**Figure 3:**
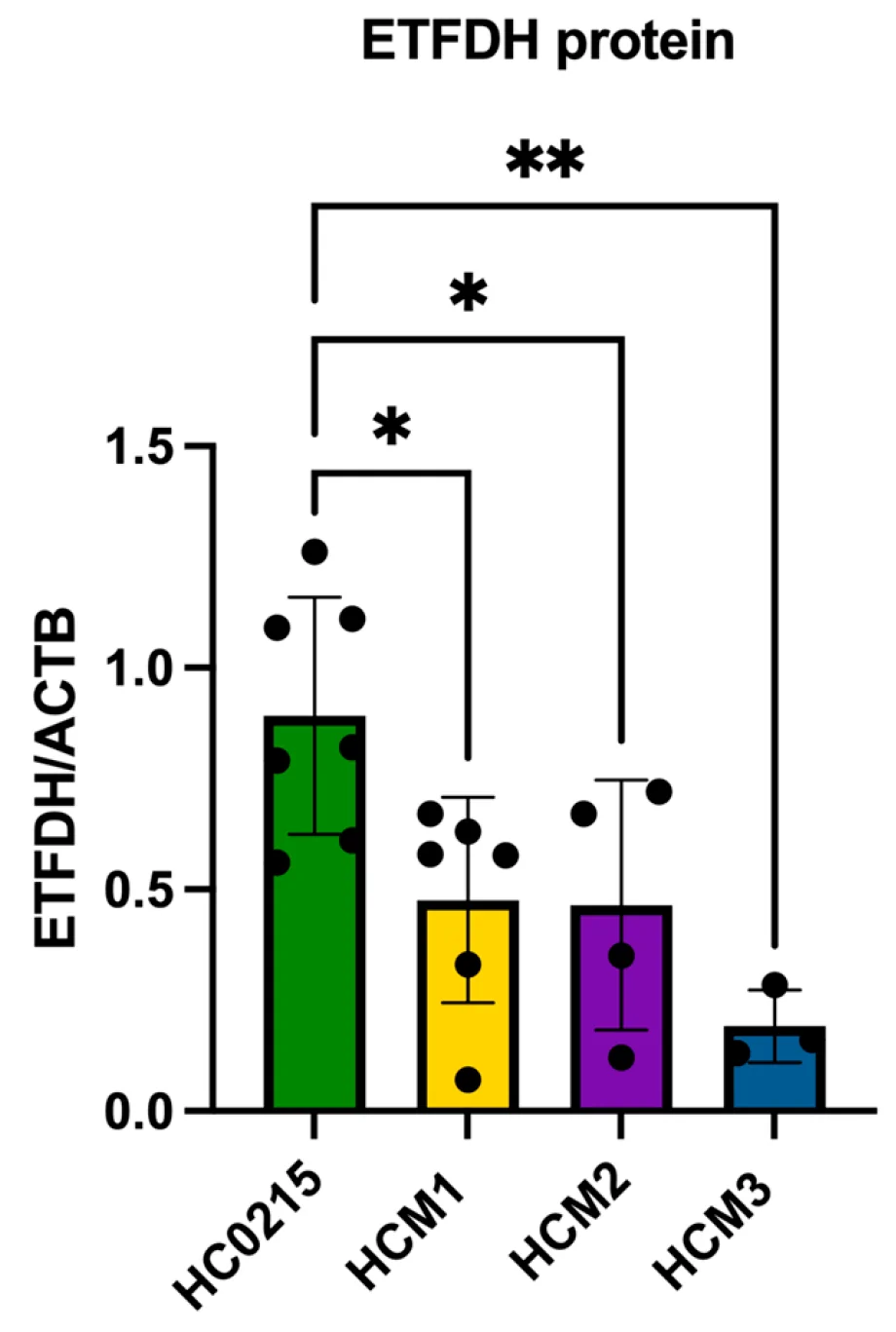
The bar graph shows ETFDH protein levels normalized to Beta Actin. Each bar represents ETFDH protein quantification of an individual: father (HCM1), children (HCM2 and HCM3), and healthy control (HC0215). Black dots indicate individual data points, and error bars represent standard deviation. All three patients show markedly lower protein levels compared to the control. (* = *p* < 0.05, ** = *p* < 0.01, ANOVA followed by Dunnett’s correction vs. HC0215).

**Figure 4:**
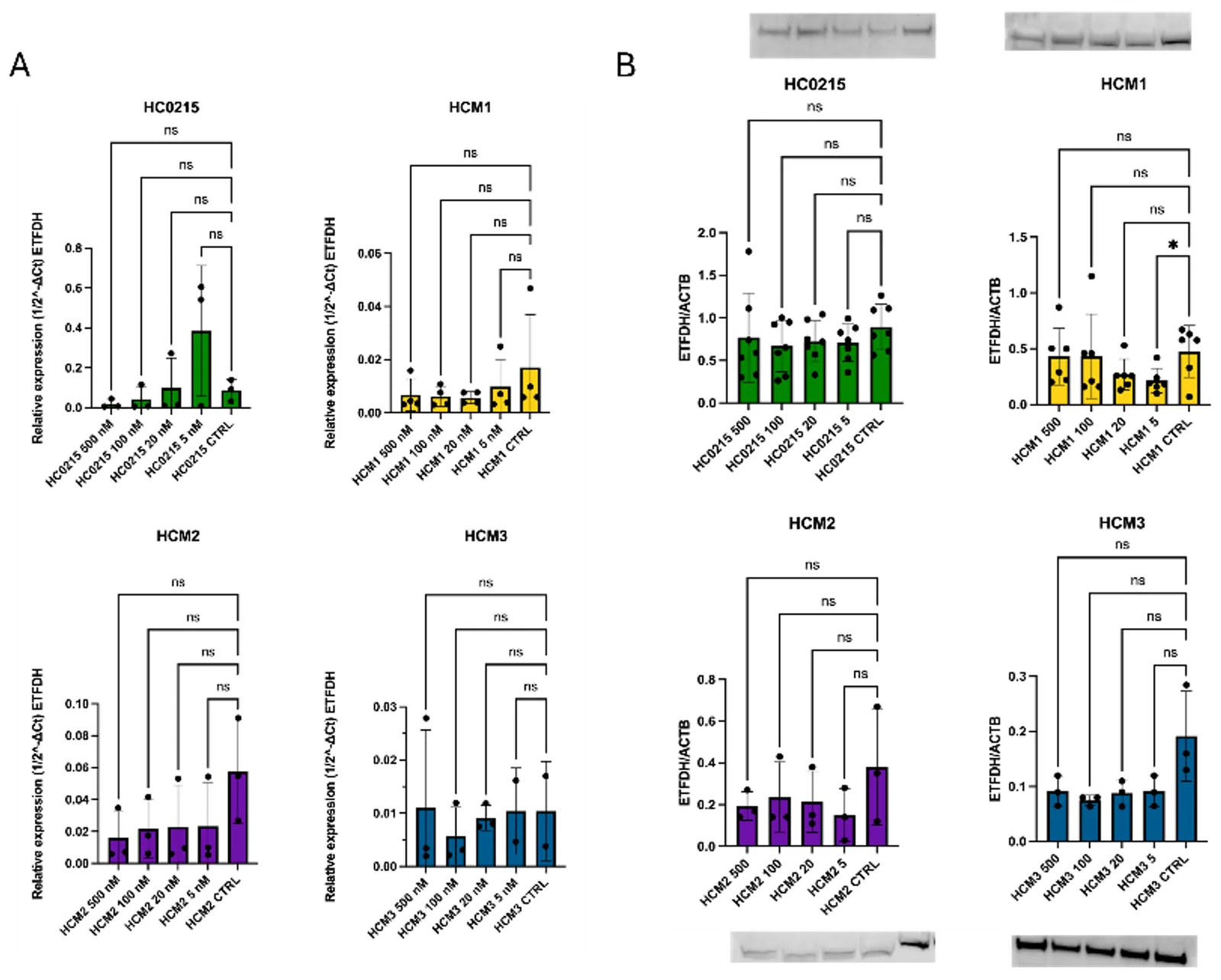
(**A**) mRNA expression analyzed by RT-qPCR of *ETFDH* in immortalized fibroblasts of patients HCM1, HCM2, and HCM3, and HC0215 healthy control after one week riboflavin treatment at different concentrations (500 nM–100 nM–20 nM–5 nM). (**B**) Protein expression of ETFDH analyzed by Western blot in immortalized fibroblasts of patients HCM1, HCM2, and HCM3, and HC0215 healthy control after one week riboflavin treatment at different concentrations (500 nM–100 nM–20 nM–5 nM) (ETFDH indicates the band for ETFDH protein, while ACT indicates bands for actin protein used to normalize expression; bands are in same order as bar graph) (* = *p* < 0.05, ANOVA followed by Dunnett’s correction vs. HC0215, ns = not significant).

Aiming to confirm the in silico observation, we sought to assess the ETFDH protein levels. Fibroblasts from the affected father (HCM1) and his two children (HCM2 and HCM3) displayed approximately 50% of ETFDH protein levels (* = *p* < 0.05; ** = *p* < 0.01, one-way ANOVA) relative to the healthy control (HC0215) (Figure 3) even under standard riboflavin conditions, despite a similar mRNA expression (Figure 4A). Decreasing riboflavin concentrations (5 nM, 20 nM, 100 nM, and 500 nM) compared to standard culture medium (~1 μM riboflavin) were used to assess a minimum dose at which treatment can restore protein control levels. Western blot analysis showed no significant changes in ETFDH protein in HCM2, HCM3, or HC0215 cells under any of the tested conditions. In contrast, in HCM1 fibroblasts, the ETFDH protein expression was significantly lower in cells treated with 5 nM riboflavin compared with standard medium (Figure 4B, * = *p* < 0.05, one-way ANOVA, Dunnett’s post-test). To assess if the effect of riboflavin on the ETFDH expression is due to the modulation of RNA levels, RT-qPCR analysis was performed on immortalized fibroblasts from the affected father (HCM1), the two siblings (HCM2 and HCM3), and a healthy control (HC0215). Cells were thus treated for one week under varying riboflavin concentrations (5 nM, 20 nM, 100 nM, and 500 nM, compared with the standard medium at ~1 µM), and assessed for ETFDH-specific mRNA expression. No statistically significant differences in ETFDH mRNA levels were observed at any of the tested riboflavin concentrations within each cell line compared with standard culture conditions (Figure 4A).

Furthermore, to determine the mitochondrial mass and thus assessing if the reduction in mitochondrial ETFDH is due to the reduction in mitochondrial number/mass, an analysis of the mitochondrial number was performed using mitoRED staining (Supplementary Figure S5). No significant changes in riboflavin-supplemented cell lines were observed, while, after riboflavin withdrawal, only HCM1 displays a significantly increased mitochondrial content.

### 2.6. Riboflavin Affects ETFDH and CIII Interactions

**Figure 5:**
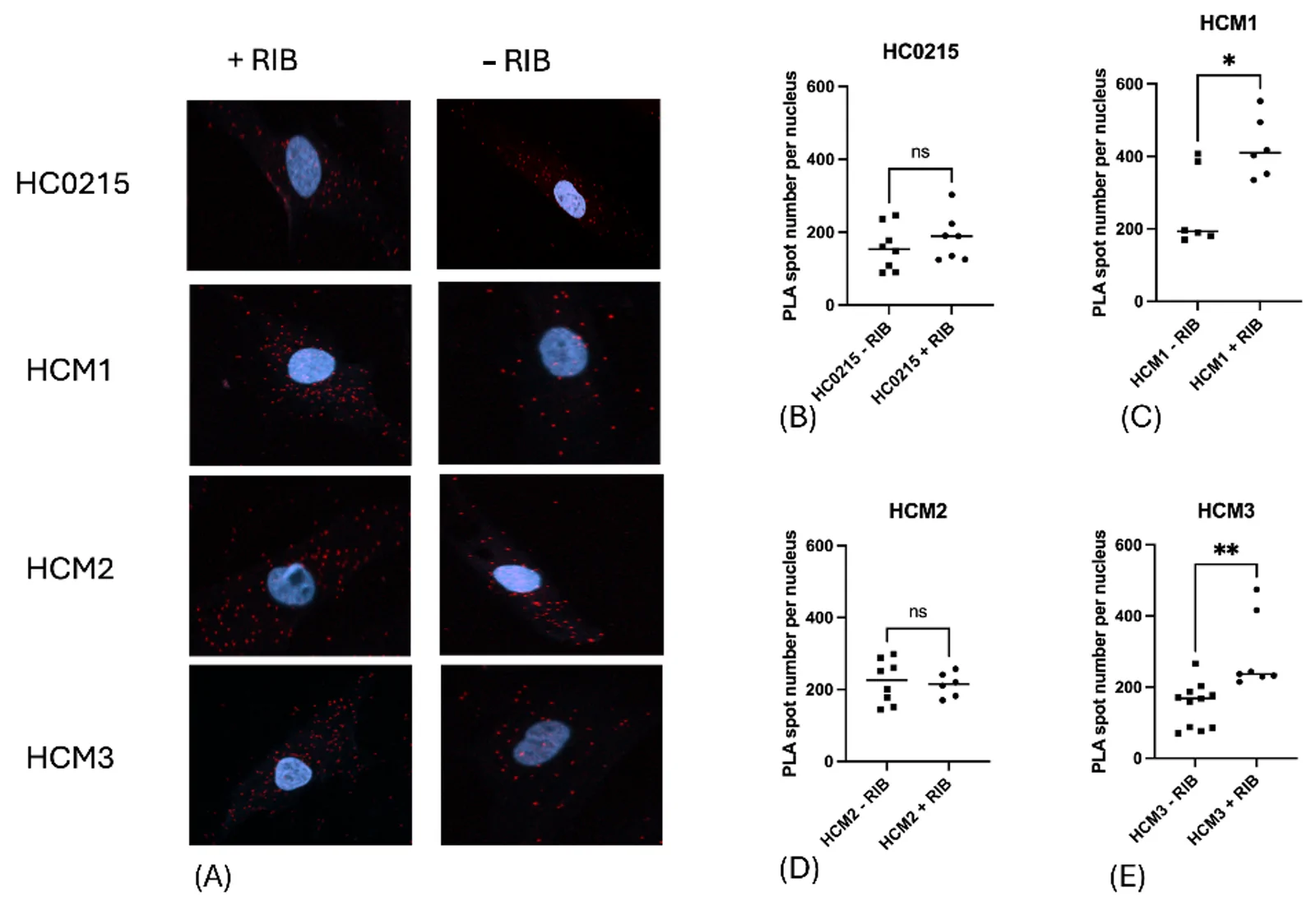
Representative images of proximity ligation assay (PLA) carried out on immortalized fibroblasts (HC0215, healthy control) after one week of culture in either riboflavin-supplemented medium (1 µM, +RIB) or under riboflavin-depleted conditions (5 nM, −RIB) are shown in (**A**) (magnification 63×). Quantification of PLA signal is presented in (**B**–**E**) (* = *p* < 0.05; ** = *p* < 0.01, unpaired *t* test, ns = not significant).

To further explore the effects of riboflavin availability on mitochondrial protein interactions, proximity ligation assay (PLA) was performed on immortalized fibroblasts cultured for one week in either riboflavin-supplemented (1 µM) or riboflavin-depleted conditions (5 nM). We sought to quantify the interaction between ETFDH and complex III. Representative images from the healthy control line (HC0215) illustrate the distribution of the fluorescent PLA signal localized near the nucleus and throughout the cytoplasm (Figure 5, left).

A quantitative analysis of the PLA signal revealed a significant reduction in the number of PLA dots per nucleus in HCM1 and HCM3 fibroblasts under riboflavin-depleted conditions compared to riboflavin-supplemented cells (* = *p* < 0.05; ** = *p* < 0.01, unpaired *t* test) (Figure 5C,E), indicating a reduction in the interaction between ETFDH and complex III. In contrast, no significant differences were observed in HCM2 or in the healthy control cell line HC0215 (Figure 5B,D). The absence of a significant difference in HCM2 is possibly due to the age of the patient, being the youngest of the family. These findings indicate a riboflavin-dependent modification of ETFDH and complex III interactions in a subset of patient-derived fibroblasts, consistent with a cell-line-specific vulnerability to flavin depletion.

### 2.7. Analysis of Mitochondrial Respiratory States

**Figure 6:**
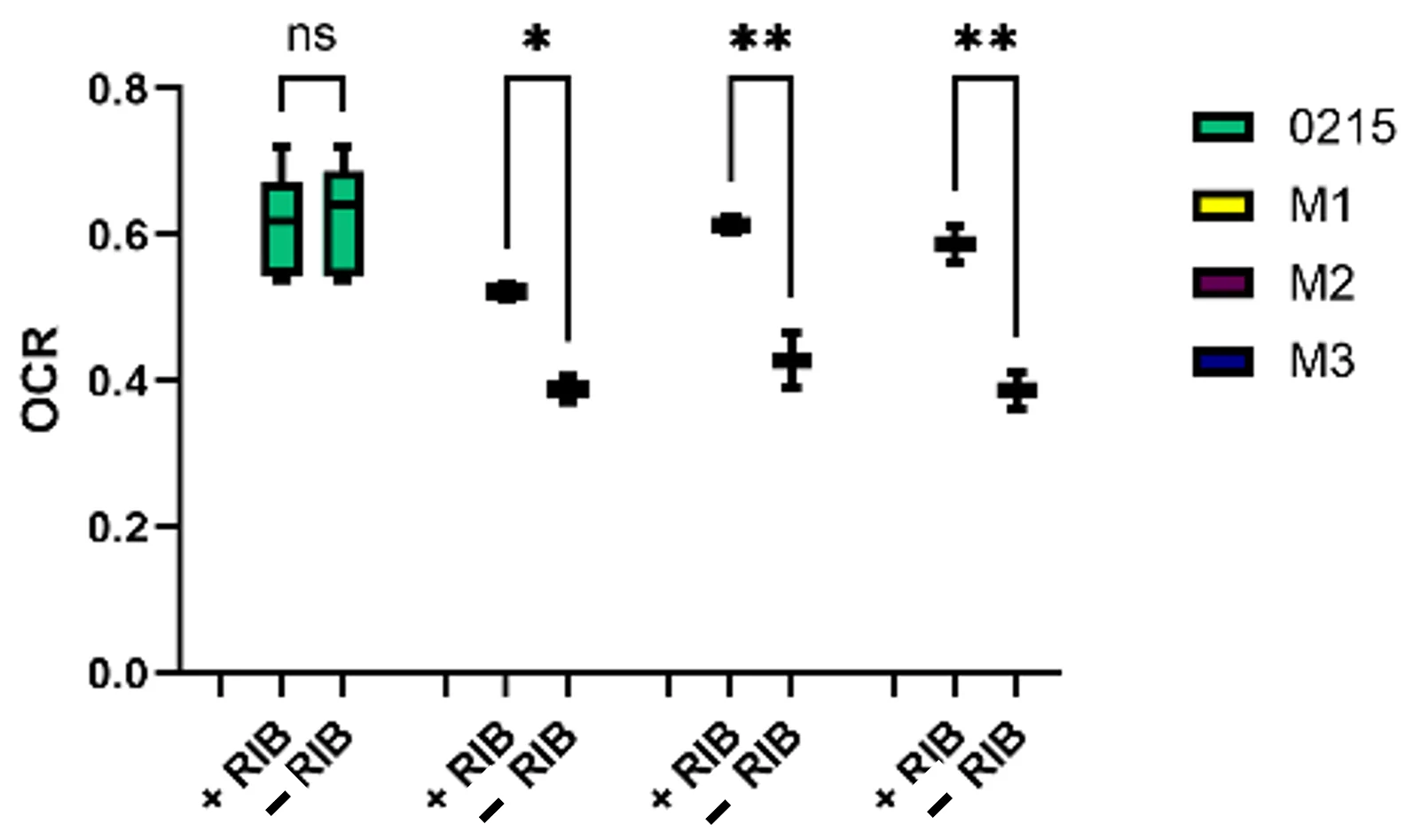
Oxygen consumption rate (OCR) after background subtraction (ambiental oxygen levels), normalized to succinate following addition of octanoyl-carnitine of immortalized fibroblasts of patients HCM1, HCM2, and HCM3, and HC0215 healthy control after one-week reduced riboflavin (5 nM, −RIB) treatment. (* = *p* < 0.05, ** = *p* < 0.01, ANOVA followed by Bonferroni correction vs. HC0215, ns = not significant).

To further assess the effect of riboflavine depletion, we decided to use HRR that permits the analysis of the respiratory state, separating them by the use of specific mitochondrial inhibitors. Using this approach, we estimate the oxygen consumption rate when octanolyl-carnitine (OCT) was used as the substrate and we found that, in low riboflavin concentrations (5 nM), the oxygen consumption was lower in respect to the standard riboflavine concentration (1 µM) in all the patients cell lines (HCM1, HCM2, and HCM3) but not in controls (HC0125) (Figure 6, * *p* < 0.05, ANOVA, Bonferroni’s correction).

## 3. Discussion

Although Multiple Acyl-CoA Dehydrogenase Deficiency (MADD) is typically considered an autosomal recessive disorder, recent data suggest that a non-negligible fraction of patients may carry only a single pathogenic ETFDH allele. In fact, a recent meta-analysis found that approximately 10% of late-onset MADD patients harbour a single heterozygous ETFDH variant with a milder phenotype compared to bi-allelic carriers, raising the possibility of more complex inheritance or non–loss-of-function mechanisms [3]. Consistently, the family described here presents a clinically significant phenotype (fatigue, high carnitine levels, and lipid accumulation in muscles) carrying only one copy of the ETFDH c.1798A>C (p.Asn600His) variant. The presence of symptoms in both the grandfather and the father—despite carrying only one mutated allele—is unexpected for a recessive disorder and strongly supports a dominant or haploinsufficient contribution of this specific variant. Furthermore, the children display an altered acylcarnitine profile, compatible with MADD, further strengthening the dominant model of inheritance. Whole genome analysis (as suggested by Zhang et al., 2025) [3] did not reveal a “hidden” variant in the ETFDH gene. Indeed, these observations could suggest a dominant mode of inheritance; however, further data (i.e., the same mutation in other families, unrelated to this one) are needed to truly confirm the dominant pattern.

At the transcript level, digital PCR demonstrated an equal distribution of wild-type and mutant alleles (50% each), indicating that no allelic imbalance occurs. However, despite the equal transcript abundance, the total ETFDH protein levels were reduced by approximately 50% or more. This discrepancy between transcript levels and protein abundance indicates that the mutation does not affect transcription, but, rather, impairs post-transcriptional processes such as folding, stability, or degradation, as previously shown for other mutations [11,12].

In the absence of clear evidence defining a functional threshold for ETFDH, it cannot be assumed that a 50% residual activity is always clinically benign; Lupica et al. identified three patients carrying a single pathogenic variation in heterozygosity, in which the protein levels were reduced [13]. Indeed, most carriers of ETFDH variants remain asymptomatic, but a subset of patients—possibly exposed to additional stressors or presenting specific variants—develop disease with different degrees of morbidity despite the heterozygosity [14]. Consistent with these previous observations, the variant found in this family possesses a high pathogenic score. Furthermore, a recent study demonstrated that ETFDH is physically associated with complex III and is required for normal CIII activity and OXPHOS efficiency in the skeletal muscle, supporting the view that ETFDH is fundamental not only in fatty acid oxidation but also as an integral component of a broader respiratory metabolon [5]. The variation described in our study lies within the C-terminal 4Fe-4S domain, which mediates the electron transfer from ETFDH to ubiquinone (UQ). This variation decreases the number of hydrogen bonds linking an unstructured loop with an α-helix, possibly destabilizing the three-dimensional architecture of the UQ-binding/4Fe-4S domain (Figure 1). Indeed, variants affecting this region have been shown to severely impair the ETFDH-mediated electron flow and protein stability [15,16]. Structural protein modelling indicated also that the Asn600His substitution likely reduces protein stability. Thus, the p.Asn600His variant may destabilize the ETFDH protein, reducing its amount and lowering its ability to interact with complex III and thus decreasing the efficiency of the fatty-acid-derived electron transfer into the respiratory chain [17]. Further work is needed to experimentally assess the protein stability and recycling, currently outside the scope of the present work.

Indeed, a previous work has demonstrated that ETFDH dysfunction can affect not only fatty acid oxidation but also UQ homeostasis and respiratory-chain activity [4,18]. It is thus possible to hypothesize also a dominant-negative mechanism, where the mutant ETFDH protein improperly binds ETF-A/B or UQ, trapping electrons and preventing efficient turnover [18]. In this case, the mutant protein may exert a dominant-negative-like effect by competing with the wild-type protein for essential cofactors or interaction partners (such as ETF, FAD, components of the ETF–ETFDH–UQ axis, or complex III), providing then another possible causative explanation for the observation that, in this family, heterozygous carriers exhibit clinical symptoms. Further work is needed to confirm this hypothesis, also assessing other genetic variants in the same domain to determine if a general mechanism is present.

Functionally, the reduced levels of total ETFDH protein were accompanied by decreased mitochondrial respiration in the FAO pathway (in the reduced riboflavin condition), while the total mitochondrial mass/number is unchanged (Supplementary Figure S5), indicating, once more, a defect in the electron chain transport from ETFDH to complex III. Moreover, mutant cells display a reduced association of ETFDH with complex III in the absence of riboflavin supplementation. These defects are consistent with the impaired electron transfer within the ETF–ETFDH–UQ axis, as indicated by the HRR results, where, in the depleted-riboflavin condition, the respiration rate is reduced in patients’ cells, while it does not change in control fibroblasts, suggesting, then, a defect in the entry of electrons’ mitochondrial respiratory chain from the fatty acid oxidation pathway. Under the conditions of limited riboflavin availability, the mitochondrial FAD is reduced and mutant ETFDH stability is impaired, as has been demonstrated by Xu et al. in other model systems [12]. Our results indicate that, when riboflavin was supplemented, mutant ETFDH rescued the interaction with complex III, accompanied by an overall improvement in cellular respiration. These effects are consistent with the known clinical response to riboflavin therapy, indicating that the biochemical defects caused by the mutation are at least partially reversible.

Interestingly, despite these molecular and bioenergetic defects, we did not observe significant differences in the mitochondrial membrane potential (Δψ) between patient fibroblasts and controls, both in the basal or under reduced riboflavin condition (Supplementary Figure S6). This observation reflects the finding detected in HRR, where only in the FAO pathway is the OCR reduced, while the total electron transfer capacity (ETC) is preserved. Indeed, ROS levels also did not show any changes (except for the father’s HCM1 cell line) (Supplementary Figure S7), indicating that the pathogenic mechanism relies only on the loss of electron flux from the FAO pathway. Together, these data support a model in which the p.Asn600His variant acts through a structurally disruptive mechanism, by reducing the total protein abundance, impairing the ETFDH function, and, ultimately, altering the mitochondrial energy metabolism even in the presence of one intact allele. This mechanistic insight may contribute to a better understanding of rare dominant presentations of riboflavin-responsive MADD and highlights the importance of evaluating the ETFDH protein stability and electron transfer efficiency in addition to genetic status alone.

## 4. Materials and Methods

### 4.1. Study Sample

The family was enrolled at the Rare Disease Unit of IRCCS Burlo Garofolo (Trieste, Italy). The study was conducted in accordance with the Declaration of Helsinki and approved by the Ethics Committee of I.R.C.C.S. “Burlo Garofolo” (protocol code 02/2024 22.02.2024 approved on 28 March 2024). Informed consent was obtained from all subjects involved in the study. It comprised a grandfather (71 years old) (I-1 in Supplementary Figure S1), father (45 years old) (II-1 in Supplementary Figure S1), both with a biochemical and histological diagnosis of MADD, and his daughter and his son (8 and 10 years old, respectively) (III-2 and III-1, respectively, in Supplementary Figure S1). Family history was carefully collected for all recruited individuals, including geographical origin and potential consanguinity. No family was found to originate from a genetic isolate or to report known consanguinity within the last three generations.

Specifically, the grandfather developed progressive muscle weakness in his thirties and was diagnosed with MADD based on muscle biopsy showing lipid accumulation and an altered blood acylcarnitine profile. At 19 years of age, the father developed muscle weakness following tonsillectomy and was subsequently diagnosed with MADD based on biochemical laboratory findings (elevated blood acylcarnitine levels) and mild lipid accumulation on muscle biopsy. In both patients, symptoms receded with riboflavin therapy (150 mg/day). Despite an altered blood acylcarnitine profile, the children were asymptomatic; nevertheless, riboflavin was administered as a preventive measure at a very early age (after weaning). All family members carried underwent Whole Exome Analysis and the only variant found linked to the phenotype is the ETFDH variant (NM_004453.4) c.1798A>C in heterozygosity.

### 4.2. Cell Culture

Primary skin fibroblasts derived from a punch biopsy from the father and the two children were cultured in DMEM high-glucose supplemented with l-glutamine, 10% fetal bovine serum (FBS) and 1% penicillin/streptomycin, and vitamin solution (MEM, EuroClone, Milan, Italy, ECM0556D). Cultures were maintained at 37 °C and 5% CO_2_ following standard procedures. Cells were immortalized using a cell immortalization kit from ABM (Lenti-hTERT Virus, ABM, Richmond, BC, Canada) and these cell lines were employed in all experiments. Cell lines identification: HCM1 from father (II-1 Supplementary Figure S1), HCM2 from daughter (III-2 Supplementary Figure S1), and HCM3 from son (III-1 Supplementary Figure S1).

### 4.3. Riboflavin Treatment

The cells were tested for the experiments in a riboflavin-depleted medium composed by a customized Minimum Essential Medium without riboflavin (ME23747, Grand Island, NY, USA) and dialyzed FBS. Different concentrations of riboflavin (R9504, Merck, Rahway, NJ, USA) were supplemented in the medium (500 nM, 100 nM, 20 nM, and 5 nM; riboflavin concentration in standard medium is 1 μM). Cells were treated with riboflavin for one week and the medium was changed twice per week. All the experiments were carried out on the 7th day of treatment.

### 4.4. Quantitative PCR

RNA from the cells was extracted using Quick RNA Micro Prep (R1050, Zymo Research, Irvine, CA, USA) following manufacturer’s instructions and quantified using NanoDrop (Implen, Munchen, Germany). Purified RNA (1 μg) was retrotranscribed into cDNA using the High-Capacity cDNA Reverse Transcription Kit (4374967, Thermo Fisher Scientific, Waltham, MA, USA) following the manufacturer’s instructions. Quantitative PCR (RT-RT-qPCR) was performed using TaqMan probes (Hs01031780 m1 ETFDH, Hs99999903 m1 ACTB) in a Thermal Cycle Real-Time System CFX Opus 96 (BIO-RAD, Hercules, CA, USA). The real-time PCR protocol consists of an initial step for 10 min at 95 °C, followed by 40 cycles of heating at 95 °C (15 s), and 60 °C (1 min). β-Actin was selected as a housekeeping gene to normalize the mRNA levels. The relative expression of mRNAs was determined with the comparative 2^−ΔΔCt^ method. All experiments were carried out in duplicate, and the reproducibility of the observations was confirmed in at least three independent experiments.

### 4.5. Whole Gene Sequencing

Sequencing of the whole gene was performed by Eurofins Genomics (Eurofins Scientific, Vimodrone, Italy). Briefly, DNA from whole blood of the father (M1) was extracted, and sent to the company. There, the DNA was amplified with specific primer for NGS sequencing (amplicon sequencing) covering the entire ETFDH sequence, and sequenced using NovaSeq (Illumina, San Diego, CA, USA). Data were subsequently analysed using Emdgene software (V35.9) (Illumina, San Diego, CA, USA).

### 4.6. Sanger Sequencing

cDNA (10 ng) was amplified by PCR using the primers Forward: 5′-AGCCGGCACACTTAACCTTA-3′ and Reverse: 5′-GAGGTAGGAAGATGCTGCATTC-3′. Reactions were carried out with a thermostable fusion polymerase (1725310, iProof HF mix, BIORAD, Hercules, CA, USA) under the following cycling conditions: initial denaturation at 98 °C for 30 s; and 29 cycles of 98 °C for 10 s, 63 °C for 30 s, and 72 °C for 15 s; followed by a final extension at 72 °C for 5 min and storage at 4 °C until use. The resulting amplicon (474 bp) was purified and subjected to Sanger sequencing according to standard protocols.

### 4.7. Digital PCR

Allelic expression of the c.1798A>C (p.Asn600His) ETFDH variant was quantified by digital PCR using 50 ng of cDNA from patient-derived fibroblasts. Digital PCR reactions were prepared according to the provided protocol: 1 μL of cDNA (~0.50 μg), 10 μL of 4× QIAcuityTM Probe PCR Master Mix (250102, Qiagen, Hilden, Germany), 2 μL of 20× primer-probe mix, and DNase/RNase-free water was added to achieve a final volume of 40 μL. The contents of each well were then transferred into the wells of a QIAcuityTM Nanoplate 26k 24-well nanoplate (250001, Hilden, Hilden, Germany), in which one reaction mix is separated into approximately 26,000 partitions. The nanoplate was sealed using the QIAcuity Nanoplate seal provided in the QIAcuity Nanoplate Kit. The PCR conditions were as follows: one cycle at 95 °C for 2 min, 40 cycle steps at 95 °C for 15 s, and 60 °C for 30 s. After PCR, the amplification target was identified by assessing the fluorescence in all positive partitions. In our experiment, the probe fluorophores were FAM (green) for mutated cDNA/HEX (yellow) for wild-type cDNA. Poisson statistics were employed to determine the average quantity of target DNA per partition. QIAcuity Software Suite 2.1.8.23 analyses the mutation frequencies in target samples as the absolute quantification of copies of each wild-type and mutant target present in the samples.

### 4.8. Immunoblot

Proteins from the cells were extracted in a lysis Ripa buffer adding a protease inhibitor (1862208, Thermo Fisher, Waltham, MA, USA). Protein lysates were fractionated by SDS–PAGE and transferred onto nitrocellulose membranes for immunoblot analysis using the iBlot 2 Dry Blotting System (IB21001, Carlsbad, CA, USA). The primary monoclonal antibody anti-ETFDH (1:200—PA5100143 Invitrogen, Carlsbad, CA, USA), anti-ACTB (1:2000—8H10D10 Cell Signalling, Danvers, MA, USA) and secondary antibodies anti-rabbit (1:500) and anti-mouse (1:2000) were incubated using the iBind device (Invitrogen). Blots were revealed using the SuperSignal™ West Pico PLUS Chemiluminescent reagent (34577, Thermo Fisher, Waltham, MA, USA), and the intensity of the bands was quantified using a ChemiDoc MP imaging system (BioRad, Hercules, CA, USA) and ImageJ 2.16 analysis software.

### 4.9. Proximity Ligation Assay

Cells were seeded at a density of 2500 cells per well and were cultured with riboflavin-supplemented medium (1 µM) or under riboflavin-depleted conditions (5 nM) for one week, changing the media twice a week in black 96-well plates (Primo Screening Plate 96 TC-treated (ECPCR0221, EuroClone, Milan, Italy), optimized for high-quality imaging). PLA experiments were performed using the Duolink In Situ Green Starter Kit Mouse/Rabbit (DUO92008 Merck, Rahway, NJ, USA) following the manufacturer’s instructions. Acquisition of the images was performed using a Zeiss LSM 900 Confocal instrument (ZEISS, Oberkochen, Germany). Images were processed using the software Zen Zeiss 3.8.

### 4.10. Mitochondrial Staining

Cells were seeded at a density of 2500 cells per well and were cultured with riboflavin-supplemented medium (1 µM) or under riboflavin-depleted conditions (5 nM) or one week, changing the media twice a week in black 96-well plates (Primo Screening Plate 96 TC-treated (ECPCR0221 EuroClone)). The following day, the medium was replaced with a 200 nM solution of MitoTracker™ Dye (53271, Merck, Rahway, NJ, USA), diluted in Hanks’ Balanced Salt Solution (HBSS, ECB4006, Euroclone, Milan, Italy) and incubated for 30 min at 37 °C. Excess dye was removed by washing the cells twice with HBSS. Nuclei were stained with a mounting medium with DAPI (P36971, Thermo Fisher, Waltham, MA, USA). Acquisition of the images was performed using a Zeiss LSM 900 Confocal instrument. Images were processed using ImageJ 2.16 analysis software.

### 4.11. Mitochondrial Membrane Potential (ΔΨM) Determination

Cells were seeded at a density of 4000 cells per well and were cultured with riboflavin-supplemented medium (1 µM) or under riboflavin-depleted conditions (5 nM) for one week, changing the media twice a week in black 96-well plates. (Primo Screening Plate 96 TC-treated (ECPCR0221, EuroClone, Milan, Italy)). JC-1 probe was used to determine the mitochondrial membrane potential (staining: 10 µM at 37 °C for 1 h, T3168, Thermo Fisher Scientific, Waltham, MA, USA), at the Cytation 5 Cell Imaging Multi-Mode Reader (Biotek, Winooski, VT, USA).

### 4.12. ROS Measurement

Intracellular reactive oxygen species (ROS) were quantified using the CellROX™ Green reagent (C10444, Thermo Fisher, Waltham, MA, USA). Cells were seeded at a density of 4000 cells per well and were cultured with riboflavin-supplemented medium (1 µM) or under riboflavin-depleted conditions (5 nM) for one week, changing the media twice a week in black 96-well plates (Primo Screening Plate 96 TC-treated, EuroClone, Milan, Italy, ECPCR0221). A positive control was generated by exposing parallel wells to 400 μM H_2_O_2_ for 2 h. After treatment, cells were incubated with 5 µM CellROX™ Green for 30 min at 37 °C, washed twice with PBS, and maintained in phenol-red-free medium. Fluorescence was measured using a Cytation 5 Cell-Imaging Multi-Mode Reader (Biotek, Winooski, VT, USA) at excitation/emission 485/520 nm. Background fluorescence (dye-only wells) was subtracted, and ROS levels were normalized to cell number using parallel Hoechst staining.

### 4.13. Measurement of Mitochondrial Respiration

Mitochondrial respiration was measured by high-resolution respirometry (HRR) using an Oroboros Nextgen O2k (Oroboros Instruments, Innsbruck, Austria). Immortalized patient-derived and control fibroblasts were cultured for one week, cultured with riboflavin-supplemented medium (1 µM) or under riboflavin-depleted conditions (5 nM). Cells were harvested, counted, and permeabilized with digitonin 1 mg/mL prior to loading; 1.5 × 10^6^ cells were added to each 2 mL chamber containing MiR05 at 37 °C, and oxygen flux was recorded with DatLab 8v. Fatty acid oxidation was assessed using the substrate–uncoupler–inhibitor titration protocol SUIT-025 (https://wiki.oroboros.at/index.php/SUIT-025_O2_mt_D057 (accessed on 6 July 2025), with the following sequence of additions (final concentrations): ADP (1 mM), malate (0.1 mM), octanoylcarnitine (0.5 mM), cytochrome c (10 µM), and antimycin A (2.5 µM). In this protocol, electrons derived from β-oxidation converge at the Q-junction from two branches: acyl-CoA dehydrogenases → ETF → ETF:ubiquinone oxidoreductase (ETFDH), and matrix-dehydrogenase-derived NADH → complex I; malate is therefore required, and complex I-linked flux contributes to the F state. Outer mitochondrial membrane integrity was verified by the cytochrome c response (±5%), and all fluxes were corrected for residual oxygen consumption determined after antimycin A. Oxygen fluxes were corrected for residual oxygen consumption and normalized to cell number (pmol O_2_·s^−1^·10^−6^ cells). To obtain an internally normalized, mitochondrial-content-independent measure, FAO-supported respiration was additionally expressed as a flux control ratio relative to succinate-supported (OXPHOS/ET) capacity measured in the same SUIT-025 run. Data are from 3 independent experiments per condition.

### 4.14. Statistical Analysis

Data were analysed using GraphPad Prism 10.2.3 (GraphPad Software LLC, San Diego, CA, USA). For multiple comparison analysis, ordinary one-way ANOVA, followed by Bonferroni or Dunnett correction was employed.

## Figures and Tables

**Table 1:** Acylcarnitine profile of the patients (M1 = Father, M2 = Sibling 2, M3 = Sibling 1) before and after riboflavin treatment (B2). The symbol \ indicates the normalization of the parameter. C* indicates the specific acylcarnitine moiety, with numbers giving the –CH3 (methyl) side chain content. The typical MADD profile shows an increase in middle-(C6-C8-C10-C10:1 and C12) and long-(C14-C14:1-C16 and C16:1) chain acylcarnitines, as shown here in underlined values. It is possible to observe that a first treatment with riboflavin (B2) does not completely normalize parameters in father (M1) and older sibling (M2), while, in younger sibling (M3), it restores normality.

	M1	B2	M2	B2	M3	B2	Normal Ranges
C0	58.71	\	54.75	\	41.06	\	19.93–52.78
C5DC + C6OH	0.26	0.22					0.04–0.11
C6	1.13	0.34					0.03–0.16
C6DC	0.44	0.18					0.03–0.13
C8	**5.07**	**1.31**	**2.33**	**0.66**	**0.93**	\	0.05–0.46
C10	**5.19**	**1.63**	**4.11**	**1.11**	**1.45**	\	0.09–0.87
C10:1	1.16	\	0.88	\	0.51	\	0.04–0.37
C12	0.56	\	0.46	\	0.13	\	0.03–0.25
C12:1	0.24	\	0.24	\			0.03–0.25
C14	0.13	\					0.01–0.05
C14:1	0.4	0.06	0.3	\			0.03–0.23
C14:2	0.11	\	0.12	\	0.06	\	0.01–0.10
C16	0.13	\					0.04–0.12
C16:1	0.05	\	0.05	\			0.01–0.04

In bold C8 and C10 the most altered acylcarnitine species.

## Data Availability

The data are available upon request due to restrictions.

## Supplementary Materials

The following supporting information can be downloaded at: https://www.mdpi.com/article/10.3390/ijms27167294/s1.

## Abbreviations

The following abbreviations are used in this manuscript:

ETFDH	Electron Transfer Flavoprotein Dehydrogenase
MADD	multiple acyl-CoA dehydrogenation deficiency
UQ	ubiquinone
FAD	flavin adenine dinucleotide
CIII	complex III
