# Supplementary material for: A Family Exhibiting Autosomal Dominant Inheritance of Multiple Acyl-Coenzyme A (CoA) Dehydrogenase Deficiency (MADD) Disease
Source: Int J Mol Sci. 2026 Aug 15;27(16):7294. doi: 10.3390/ijms27167294 (PMC13513812; doi:10.3390/ijms27167294)
Supplement: Supplementary file 1 [file ijms-27-07294-s001.zip › ijms-4386603-supplementary.pdf]

Supplementary

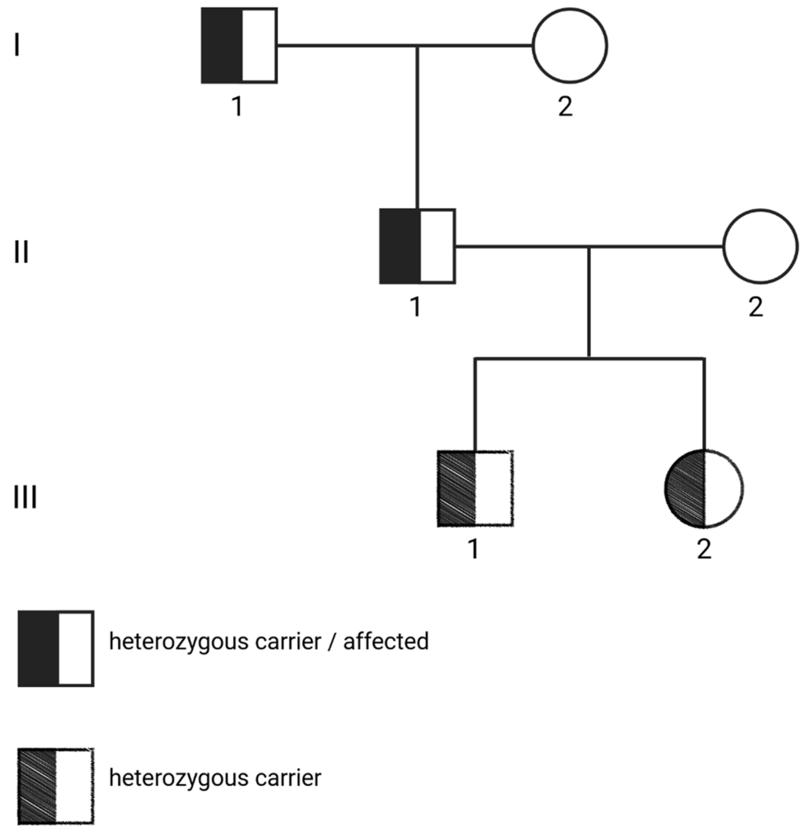

Figure S1. Pedigree of the family with multiple acyl-CoA dehydrogenation deficiency, white: unaffected subjects, black/white solid: heterozygous subjects affected, black/white striped:heterozygous subjects. I-1: grandfather (affected); II-1: father (affected) III-1, III-2: son and daughter of II-1, heterozygous carriers. Created in BioRender. https://BioRender.com/c4dihv7.

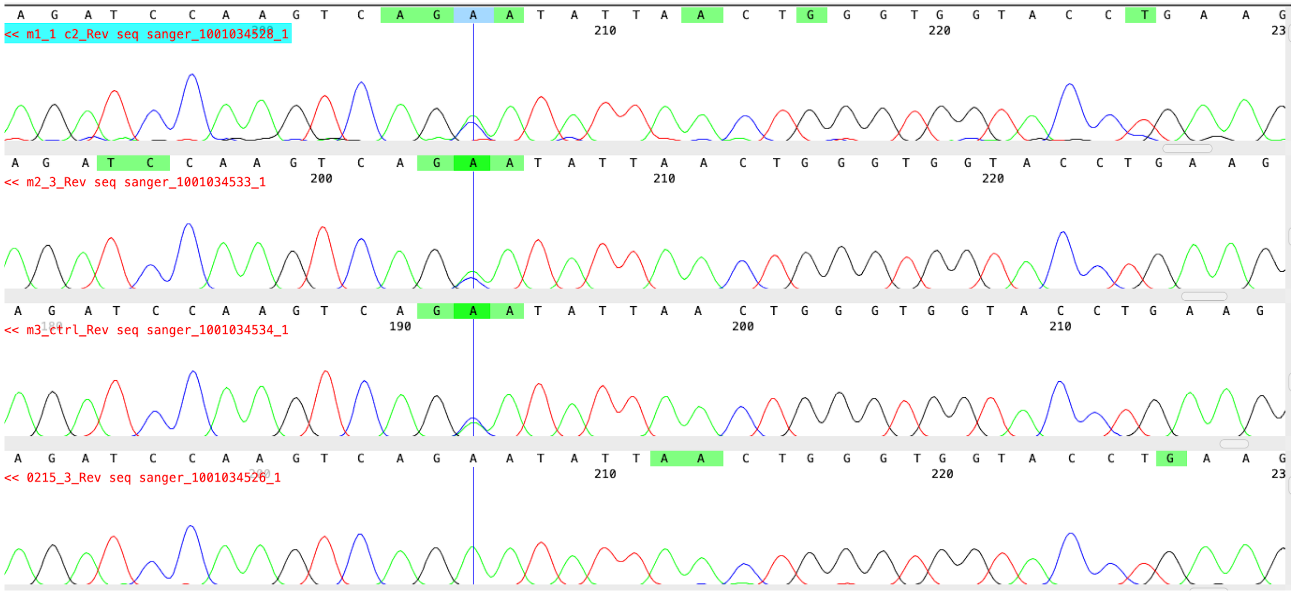

Figure S2. Sanger sequencing of ETFDH cDNA from patient-derived fibroblasts. The heterozygous c.1798A>C (p.Asn600His) variant is present in the father (M1) and his two children (M2 and M3), and absent in a healthy control (HC0215). Electropherograms illustrate the A>C substitution at position 1798.

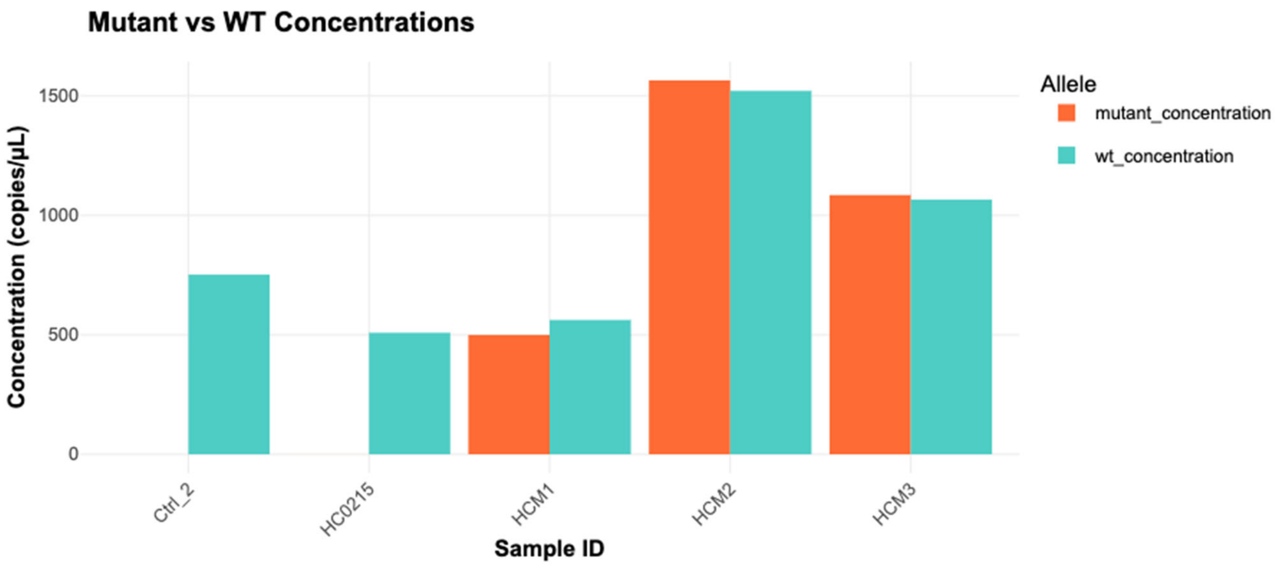

Figure S3. Digital PCR quantification of wild-type and mutant ETFDH transcripts in fibroblasts-derived cDNA. . The bar graph shows the absolute quantification (copies/µL) of ETFDH wild-type (HEX channel) and mutant c.1798A>C (FAM channel) alleles measured by digital PCR (QIAcuity, Qiagen). The father (HCM1), son (HCM2), and daughter (HCM3)

display an approximately equal concentration (~50% each) of wild-type and mutant transcripts, consistent with heterozygous status , demonstrating the absence of allelic imbalance. Healthy controls (HC0215 and Ctrl_2), expresses only the wild-type allele. Concentrations were calculated using Poisson statistics. Data analysis and visualization were performed using dPCR Analyzer v1.0, an in-house R/Shiny application developed with the assistance of AI tools (Claude, Anthropic).

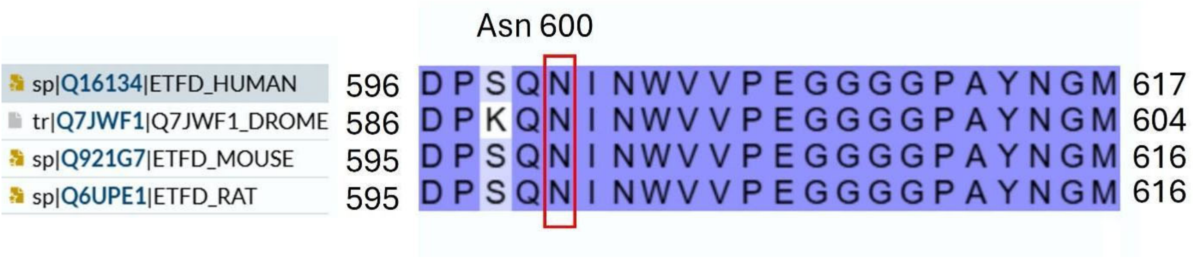

Figure S4. Asn 600 aminoacid highly conserved in human, mouse, rat and drosophila, from Uniprot website alignment tool (www.uniprot.org).

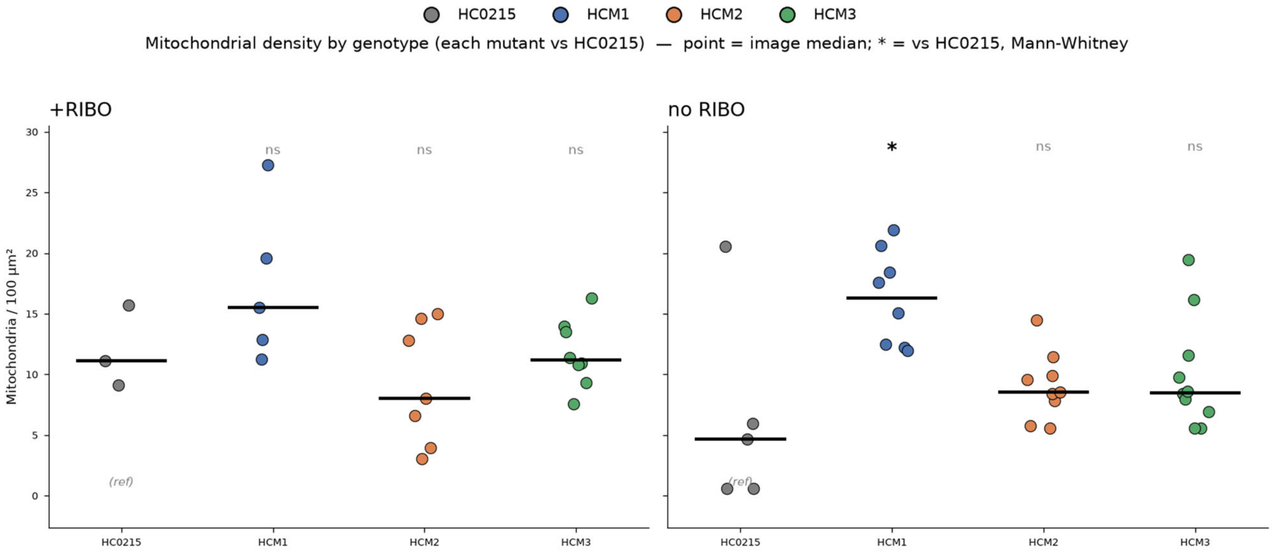

Figure S5. Mitochondrial number analysis (per cell density) showing no significant changes between the different cell lines, either with or without riboflavin; only HCM1 shows an increased mitochondrial content after riboflavin depletion

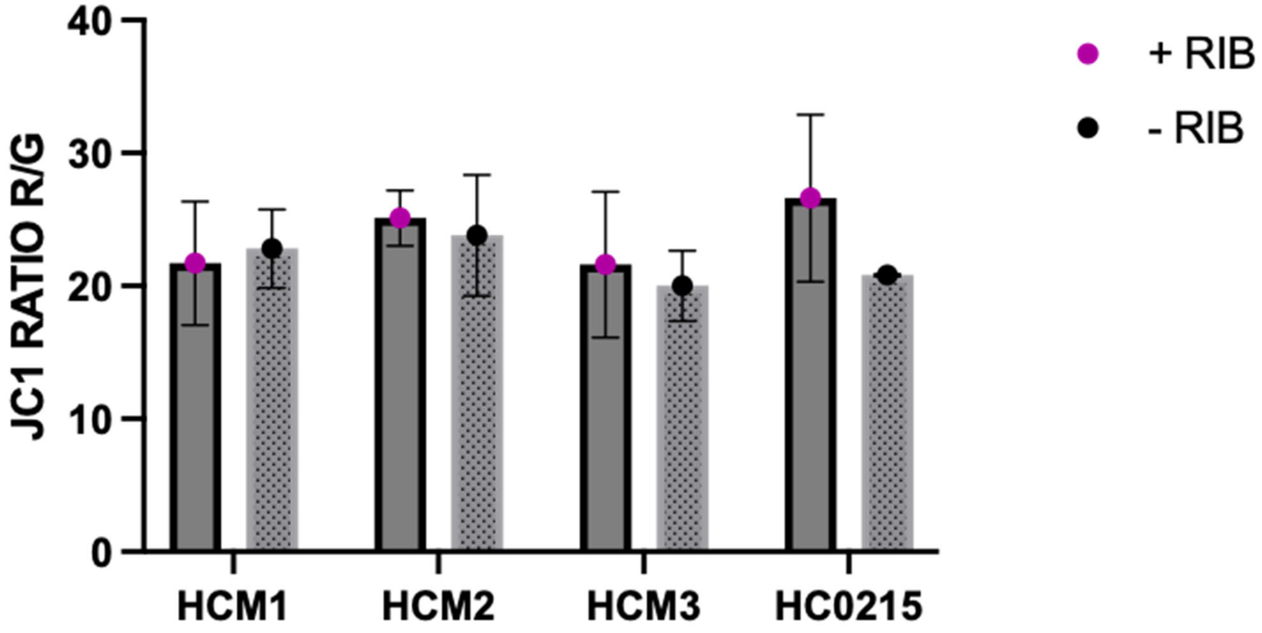

Figure S6. Mitochondrial membrane potential in immortalized fibroblast cells (HC0215, healthy control) after one week of culture in riboflavin-supplemented (+ RIB, 1 μM) or riboflavin-depleted medium (- RIB, 5 nM), analysed using the JC-1 assay. The mitochondrial membrane potential is expressed as the red/green fluorescence ratio (R/G). The data are presented as mean ± SD.

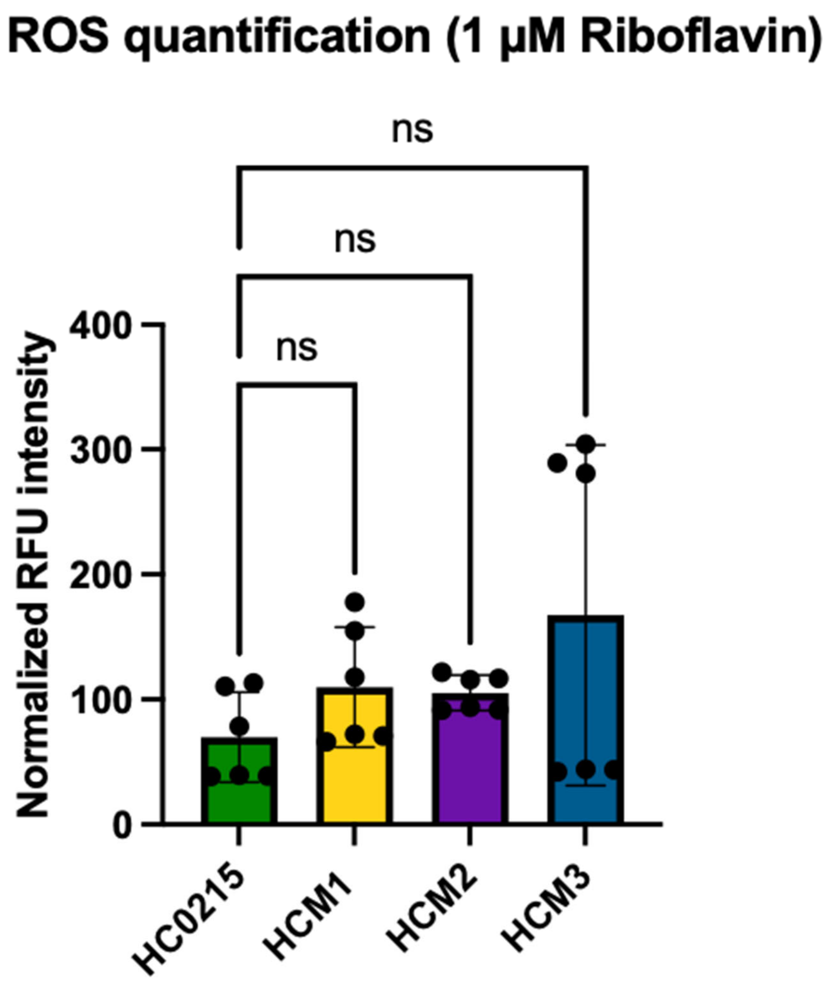

Figure S7. ROS production measured with CellROX Green Reagent. Quantitative analysis of ROS in healthy control fibroblasts (HC0215) vs patients HCM1, HCM2, and HCM3 fibroblasts normalized per number of nuclei. Analysis ANOVA one way. The data are presented as mean ± SD.

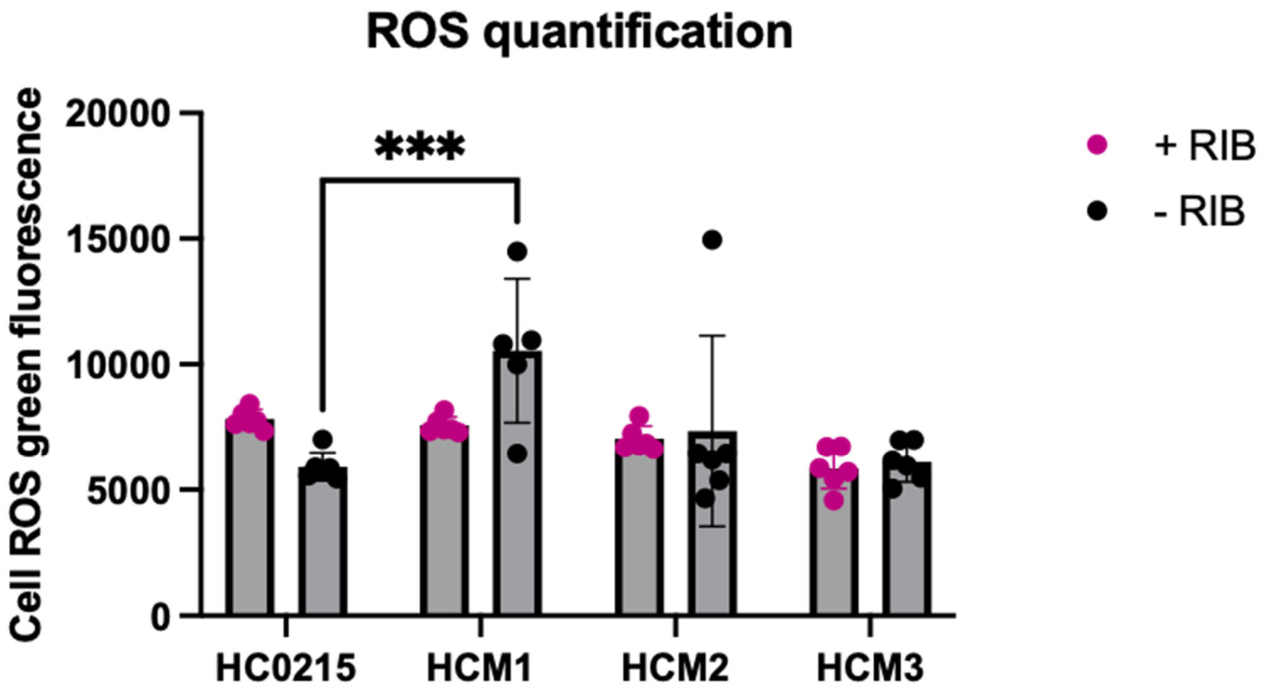

Figure S8. ROS production measured with CellROX Green Reagent. Quantitative analysis of ROS in healthy control (HC0215) vs patients HCM1, HCM2, and HCM3 fibroblasts cultured in depleted-riboflavine medium (-RIB, 5nM) or in normal conditions (+ RIB, 1 µM) normalized per number of nuclei. Analysis ANOVA two-way (Dunnett's post test, *** $p < 0.001$). The data are presented as mean ± SD.
